# Change in Negative Affective Bias following a Single Ketamine Treatment for Treatment-Resistant Depression

**DOI:** 10.1155/2023/3371272

**Published:** 2023-08-19

**Authors:** Anna J. Harvey, Stevan Nikolin, Nicholas Chand, William Flanney, Liyi Tan, Adriano Moffa, Colleen K. Loo, Donel M. Martin

**Affiliations:** ^1^University of New South Wales, Sydney, NSW, Australia; ^2^Black Dog Institute, Sydney, NSW, Australia; ^3^The George Institute for Global Health, Sydney, NSW, Australia

## Abstract

Ketamine has recently emerged as a highly effective new treatment for people with treatment-resistant depression with rapid antidepressant effects. However, these effects are often short lasting, and the potential cognitive mechanisms underlying the therapeutic effects, such as effects on emotional processing bias, remain poorly understood. In the present study, we explored potential changes in emotional and cognitive processing following a single treatment of subcutaneous ketamine in a randomised double-blind controlled study with an active control. Participants with treatment-resistant major depressive disorder (MDD) were recruited from a single site from the Ketamine for Adult Depression Study (KADS Trial) and were randomly assigned to receive racemic ketamine hydrochloride (*n* = 10) or midazolam hydrochloride (*n* = 11) in a 1 : 1 ratio. A healthy control sample (*n* = 23) was recruited to attend a single experimental session without any treatment. All MDD participants completed mood ratings and cognitive assessments prior to and one day after a single randomised treatment. The results showed no significant differences in performance changes after treatment across the majority of emotion-related (i.e., Emotional Stroop Task, Affective Go/No-Go Task) and cognitive (Ruff 2 and 7 Selective Attention Test, Controlled Word Association Test) outcome measures. Participants who received ketamine showed a significant improvement in a negative processing bias test (i.e., The Scrambled Sentence Task; Cohen's *d* = .67, *p* = .016), which was not significantly associated with improvement in psychological symptoms (*r* = −.662, *p* = .074). The results from this exploratory study suggest that a single ketamine treatment may modulate negative affective bias. Limitations to this study included the small sample size and lack of follow-up. Future larger trials are required to confirm this finding.

## 1. Introduction

Major depressive disorder (MDD) is a highly prevalent and disabling mental disorder characterised by persistent low mood and dysfunctional cognitive processing. Approximately one-third of patients with MDD suffer from treatment-resistant depression (TRD) and do not show substantial clinical improvement despite multiple courses of consecutive antidepressant treatment [1]. TRD is associated with lower health-related quality of life, increased economic burden [2], and, most concerningly, with a high risk of suicide [3]. This emphasises the importance of the development of rapidly acting treatment strategies for patients unable to respond to conventional therapies. Ketamine, an N-methyl-D-aspartate (NMDA) receptor antagonist and glutamatergic modulator, has recently emerged as a highly effective new treatment for people with TRD with rapid antidepressant effects [4]. This study sought to improve our understanding of the mechanisms underlying the rapid antidepressant effects of ketamine.

Ketamine is a racemic mixture comprising of (S)-ketamine (esketamine) and (R)-ketamine (arketamine) enantiomers. Both appear to be safe, well tolerated, and have rapid antidepressant effects [5–7]. Due to its pharmacokinetic characteristic, ketamine can be administered in several ways in TRD, including intravenous (IV; e.g., [8]), intramuscular (IM; e.g., [9]), intranasal (IN; e.g., [10]), sublingual (e.g., [11]), subcutaneous (SC; e.g., [12]), and oral (e.g., [13]). Promising findings associated with intranasal (IN) esketamine in depression (e.g., [10, 14, 15]) resulted in its approval for the treatment of TRD by the Food and Drug Administration (FDA) in 2019. A recent systematic review and meta-analysis of 19 studies [16] evaluated the efficacy of ketamine in TRD over time. Results showed depression scores decreased at 4 hours following a single ketamine infusion. Participants who received ketamine also showed more favorable clinical response and remission rates than those who received placebo, including infusions of saline solution or midazolam. The findings, however, also indicated that these effects diminished with time, 7 days posttreatment, despite the use of various dosing parameters and routes of administration [16]. Therefore, it is crucial to investigate enhanced methods of ketamine administration to further optimise patient outcomes.

With major depression, emotional regulation deficits, including negative affective biases, are considered to play a key role in the development and maintenance of the disorder [17–19]. It has been shown that these deficits predict the subsequent severity of depressive symptoms (e.g., [20, 21]) and that they manifest more strongly at the most severe levels of depression [22]. Poor emotion regulation has been hypothesised to be related to maladaptive strategy use, particularly for rumination (i.e., the focus on negative affective states), and suppression, or inhibition of the effects of external cues [18]. Examples include a slower processing of negatively valenced stimuli [23] and difficulties in stopping or inhibiting the processing of negative material [24], or a negative processing bias. For instance, on the Emotional Stroop Task, people with MDD were found to have slower response times compared to healthy controls when negative words were used, and larger interference effects for negative words compared with positive words [25]. Similarly, on the Scrambled Sentence Task (SST; [26]), individuals reporting subclinical and clinical symptoms of depression were more likely to unscramble the sentences using negative words than healthy individuals [27–29]. Negative processing bias, as indicated by high scores on the SST, has also been found to predict subsequent depression symptoms measured 4 to 6 weeks post-SST administration, even when controlling for concurrent and past depression [27].

Recent studies investigating emotional regulation deficits in people with TRD have found that a single injection of ketamine can be efficacious in reducing rumination [30] and cause sustained improvement in negative self-schema [31]. A functional neuroimaging study suggested that ketamine normalises brain functioning in MDD participants during emotionally valenced attentional processing to a similar pattern of brain activity as observed in healthy controls [32]. That study observed an interaction effect between emotion valence and mood rating scores in MDD participants following ketamine, whereby positive changes in mood scores were associated with an increased response to positive stimuli in emotional processing regions. Further, in a recent observational case-controlled study, Bottemanne et al. [33] investigated ketamine effects on belief-updating biases in 26 patients with TRD. The results showed that a single ketamine infusion strengthened optimism biases in TRD patients as they updated their beliefs more after good than bad news. These findings thus suggested that affective bias might be acutely affected by ketamine. However, since the study was not placebo-controlled, researchers failed to capture the value of specific therapeutic benefits of ketamine. Further, results were limited to performance on a belief-updating task. Assessments conducted using other emotional processing tasks as well as nonemotion-based cognitive tasks would increase our understanding of early ketamine effects on emotional regulation deficits in TRD.

In the present study, we aimed to explore potential changes in emotional and cognitive processing following a single treatment of subcutaneous ketamine in people with TRD in a randomised double-blind controlled study with an active control. This was an exploratory substudy to the larger KADS trial. A separate sample of age and gender-matched healthy controls was further recruited for comparison. We hypothesised that following a single ketamine treatment, there would be a change in negative bias processing.

## 2. Methods

### 2.1. Participants

Participants were recruited from a single site from the Ketamine for Adult Depression Study (KADS Trial: [34]), a multicentre double-blind randomised controlled trial which investigated the efficacy of repeated subcutaneous ketamine injections for people with treatment-resistant depression (TRD), trial registration ACTRN12616001096448. The protocol for this trial is available at 10.17605/OSF.IO/6FPGU. Briefly, the main inclusion criteria were aged ≥ 18 years; major depressive disorder (MDD) of at least 3 months' duration as assessed by an experienced site clinician and confirmed by the Structured Clinical Interview for DSM-5 Research Version; insufficient response to at least two adequate trials of antidepressant medications as defined by the Massachusetts General Hospital Antidepressant Treatment Response Questionnaire [35]; any concurrent antidepressant medication had to be at stable dosage for at least 4 weeks prior to and during the 4-week RCT treatment period; and score ≥ 20 on the Montgomery–Åsberg Depression Rating Scale (MADRS: [36]). Exclusion criteria were current or past psychotic disorder, bipolar disorder, disorder other than MDD judged to be the primary presenting condition, significant acute risk of suicide, substance abuse or dependence in the previous 6 months or ketamine treatment in the last 3 months, any lifetime abuse of ketamine or phencyclidine, pregnancy, and medical contraindication to the use of ketamine (Ketamine Screening Safety Tool, KSET; [37]) or midazolam. A separate sample of healthy participants was recruited from the community via study advertisements to complete the emotional and cognitive-based assessments at a single session. Exclusion criteria for the healthy sample were neurological condition or current psychiatric disorder, history of seizure or stroke, current history of drug or alcohol abuse or dependence, concurrent medication likely to affect mental performance, and history of serious head injury within the previous 12 months. Healthy controls were matched to MDD participants so that each pair was of the same gender and within five years of age. According to the Declaration of Helsinki, all participants gave informed consent to participate in the study. The study was approved by the human research ethics committee of the University of New South Wales.

### 2.2. Study Design

In this prospective study, participants with TRD were consecutively recruited from a single site of the KADS trial (no other sites were involved) and invited to attend two experimental sessions, first at pretreatment and the second one day after the first randomised treatment with subcutaneous racemic ketamine or midazolam. Participants were randomly assigned to receive racemic ketamine hydrochloride (100 mg/mL: 0·5 mg/kg) or midazolam hydrochloride (0·025 mg/kg) in a 1 : 1 ratio. Midazolam, a benzodiazepine medication, has been shown to produce short-term adverse acute cognitive effects which typically resolve within approximately one to two hours [38]. Both drugs were clear solutions for injection, presented in identical vials. A trial statistician computer-generated a permuted-block randomisation sequence (blocks were a random mixture of size two and four). Treatment allocation was sequential. Participants, mood raters, and cognitive test administrators were blinded to treatment allocation. Prior to commencing testing, all participants first completed the Depression Anxiety Stress Scale (DASS-21: [39]), a self-rating scale which assesses depressive, anxiety, and stress symptoms. Depressed participants were also assessed by blinded raters using the MADRS at pretreatment. The below tests were then administered by a trained investigator in the following order: Emotional Stroop Task, Verbal Fluency, Affective Go/No Go, Ruff 2 and 7 Selective Attention Task, and Scrambled Sentence Task.

### 2.3. Cognitive Assessment

#### 2.3.1. Scrambled Sentence Task (SST)

The Scrambled Sentence Task was administered to assess negative cognitive bias [40]. Participants were asked to unscramble as many sentences as possible from 20 trials of scrambled words into grammatically correct sentences within a four-minute time limit. The sentences consisted of six words in a random order, of which five had to be used to form a sentence. Participants were given a six-digit number to remember whilst they completed the task [26]. The outcome was the percentage of negative valence unscrambled sentences from the total of correct completed sentences.

#### 2.3.2. Affective Go/No Go (AGNG) Task

The AGNG Task (Cambridge Cognition Ltd) was additionally administered to assess affective bias. The tasks consisted of 10 blocks with rapidly presented positive and negative valence words. Each block included 18 words. At the beginning of each block, participants were given a target value (positive or negative) and were asked to push a button on a press pad as soon as they saw a word that matched the respective valence. Participants were randomised into two groups, one commencing with positive targets and the other with negative targets. The key outcomes were the response latencies (in ms) in blocks where the target valence had shifted from negative to positive or positive to negative.

#### 2.3.3. Emotional Stroop Task

The Emotional Stroop Task assesses response inhibition in the context of affective stimuli. The task was administered using Inquisit 4 (Millisecond Software). For the task, 25 words were presented in random order and color in each of five categories (positive, negative, aggressive, neutral, and color) [41]. Words could be either written in red, green, blue, or yellow. The primary outcomes were the mean response time for positive and negative words subtracted from the mean response time for neutral words (ms).

#### 2.3.4. Ruff 2 and 7 Selective Attention Test

The Ruff 2 and 7 Selective Attention Test [42] assesses concentration and selective attention. Participants were required to cross out 2 s and 7 s as quickly as possible without making mistakes. The task consisted of 20 blocks, each containing 3 lines. Each line had 10 targets and 40 distractors. The task outcome was total speed T-score, which reflects the total accurate identifications which was adjusted based on age and education normative data.

#### 2.3.5. Controlled Word Association Test (COWAT)

Verbal fluency was assessed using the Controlled Oral Word Association Test (COWAT: [43]). This task requires participants to list as many words within one minute starting with a given letter. Two different versions of the task were used, one version that used the letters F, A, and S and one version that used the letters C, F, and L, which were randomised between participants. The primary outcome was the total number of correct words.

### 2.4. Statistical Analysis

Data was analysed with the statistical software SPSS Statistics for Windows Version 26.0 (IBM Corp). Analysis of variance (ANOVA) and the *χ*^2^ test compared baseline demographic differences between the three groups. Missing data from computer malfunction or experimental error was excluded from the analysis. Repeated measure analyses of variance (RMANOVAs) were used to examine for changes in mood and performance on emotional processing and nonemotion cognitive tasks following ketamine or midazolam, with time (pretreatment and post-1 treatment) as the repeated factor and condition (ketamine and midazolam) as the between-subject factor. Post hoc tests were conducted if the time × condition interaction effect reached statistical significance. Independent sample *t*-tests were used to compare posttreatment performance with healthy controls. Exploratory Pearson's correlations are examined for associations between cognitive changes and psychological symptoms following a single ketamine treatment. Statistical significance was set at *p* < 0.05.

## 3. Results

### 3.1. Participants

Demographic and clinical data for all groups are presented in Table 1. No significant differences were found between groups for any baseline demographic factors between the three groups.

### 3.2. Effects of a Single Ketamine or Midazolam Treatment on Psychological Symptoms

Results from the RMANOVA showed significant time effects for DASS-21 Total (*F*(1.18) = 20.93, *p* < .001); DASS-21 Depression (*F*(1.18) = 19.47, *p* < .001), and DASS-21 Anxiety (*F*(1.18) = 6.69, *p* = .019). A significant time × Group interaction was found only for DASS-21 Total (*F*(1.18) = 8.81, *p* = .008). Post hoc testing revealed that participants in the ketamine group improved DASS-21 total scores after treatment (*p* < .001).

### 3.3. Neurocognitive Changes following a Single Ketamine or Midazolam Treatment

**Figure 1:**
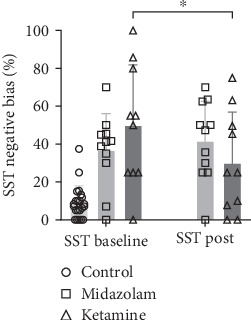
SST performance changes following midazolam and ketamine treatment. Bars show means and error bars show standard deviations. ⁣^∗^ shows a significant change in ketamine group.

Results from the RMANOVAs examining neurocognitive outcomes are shown in Table 2. Significant time effects were found for COWAT (*F*(1.18) = 4.675, *p* = .044) and Ruff 2 and 7 Total Speed (*F*(1.17) = 8.367, *p* = .010). A significant group effect was found for COWAT only (*F*(1.18) = 6.136, *p* = .023), showing overall better performance in the midazolam group. A significant Time × Group interaction was found only for the SST (*F*(1.19) = 5.728, *p* = .027), see Figure 1. Post hoc testing revealed that participants in the ketamine group performed significantly better on the SST task after treatment (Cohen's *d* = .67, *p* = .016). There were no other significant interactions for any of the remaining neurocognitive outcomes. An independent samples t-test revealed that the control group performed significantly better on the SST task when compared with the ketamine group post treatment (*t*(30) = −5.68, *p* < .001, *d* = −2.17).

### 3.4. Association between Mood and Cognitive Changes following a Single Ketamine Treatment

**Figure 2:**
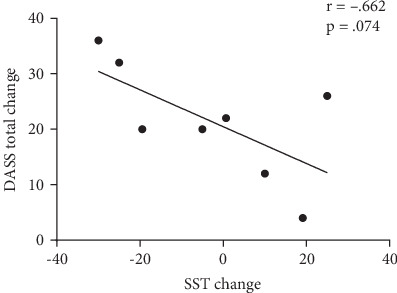
Association between change in psychological symptoms and change in the negative affective bias following a single ketamine treatment. Change scores are the differences between baseline and posttreatment scores.

The correlation between SST change and DASS-21 Total Score change did not reach statistical significance (*r*(8) = −.662, *p* = .074] (Figure 2).

## 4. Discussion

The current study investigated changes in emotional and cognitive processing following a single treatment of subcutaneous ketamine in people with TRD in a small substudy from the KADS trial. The results showed no significant differences in performance across the majority of outcome measures. Participants in the ketamine group significantly improved in the negative affective bias on the SST following a single treatment.

The current preliminary results on the Scrambled Sentence Task following a single ketamine treatment are in line with a previous study which similarly showed that a single ketamine infusion strengthened optimism biases in TRD [33]. The current study extended this work by comparing the effects of ketamine treatment with the effects of an active control (a single midazolam treatment) on the performance of other common emotion-based tasks, including those assessing negative processing bias, as well as nonemotion-based cognitive tasks. As the cognitive and mood effects of midazolam typically resolve within two hours [38], it is unlikely that any potential effects of midazolam confounded this result. The results thus further demonstrated the specificity of the effects of ketamine on negative processing bias as assessed using the SST.

The significant results for SST may be related to the characteristics of this task. During the task, participants had the freedom to unscramble the sentences using negative over positive words to reveal their negative affective bias. Research on SST shows that individuals reporting subclinical and clinical symptoms of depression are more likely to select negative over positive words compared to healthy individuals [27–29]. Other tasks which assess emotion regulation deficits (i.e., Emotional Stroop and AGNG tasks) do not involve an active choice between negative and positive stimuli, which might play a role in disclosing negative processing bias. Another potentially relevant factor was the cognitive load requirement (i.e., remembering a six-digit number while doing the task). Prior studies have found that MDD is associated with difficulties in suppressing negative stimuli from entering working memory [44–46]. It is possible, then, that with the rapid mood improvement with ketamine, working memory capacity may have improved, leading to enhanced response inhibition for the negative stimuli.

Analyses, which investigated the effects of a single ketamine or midazolam treatment on clinical symptoms, showed that participants in the ketamine group significantly improved self-reported psychological symptoms after the treatment. This is consistent with previous findings reported in meta-analyses by Romeo et al. [47] and Marcantoni et al. [16], which show that, compared with placebo (infusions of saline solution or midazolam), a single dose of ketamine significantly improved mood in TRD.

Interestingly, there was indication that change in negative affective bias following a single ketamine treatment was associated with improvement in psychological symptoms, although this effect did not reach statistical significance. DASS-21 Total assesses self-reported depressive symptoms as well as anxiety and stress symptoms. In addition to having rapid acute effects on improving mood, single treatment of ketamine has also been associated with improved anxiety symptoms (e.g. [48, 49]). Previous research of SST in depressed patients indicated that negative interpretation bias is associated with the severity of depressive symptoms [50]. Our current results suggest that improvement in negative processing bias might also be associated with generalised clinical improvement, as the DASS also assesses anxiety and stress symptoms. Interestingly, previous studies have shown that a single ketamine infusion [51, 52] as well as repeated infusions [7, 53] rapidly reduces levels of anhedonia in TRD. Anhedonia, diminished subjective experience of pleasure, was found to be significantly correlated with biases towards negative experience and away from positive experience as well as with memory for fewer positive words and more negative words in patients with depression [54]. It is possible, then, that the rapid change in negative processing bias with ketamine is related to the rapid improvement in anhedonia also observed with ketamine in depression. Mathews and Barch [55] suggested that anhedonia might modify cognitive processing for emotional information in such a way that positive information is more difficult to retrieve and sustain than negative information. Future research is required to determine whether improved psychological symptoms or reduced levels of anhedonia cause changes in negative processing bias or whether changes in negative processing bias may have a positive impact on clinical symptoms and anhedonia.

The current study showed a significant effect of a single ketamine treatment on negative cognitive bias. There were, however, several limitations to this study. First, the TRD sample size was small due to recruitment being limited to a single site of the KADS trial. These preliminary findings, therefore, require confirmation in larger trials. Additionally, most participants randomised to ketamine had concurrent ongoing antidepressant treatment, which could also have influenced their negative affective bias. Harmer et al. [56] showed that emotional processing can be regulated with antidepressant drugs in depressed patients. It is important to note, though, that these participants had been on a stable dosage of medications for at least 4 weeks, met the criteria for treatment-resistant depression, and were currently depressed when entering the study. Moreover, in the present study, there was no follow-up, so it cannot be determined how long the reduction in negative affective bias lasts. Future studies are needed to address these outstanding gaps in knowledge. For example, it needs to be determined if the positive bias is maintained following a single ketamine infusion or if these effects diminish with time, as does psychological improvement. Psychotherapy has been shown to sustain the effects of ketamine on symptoms of depression in TRD [57]. It is possible, then, that adding psychotherapy early during ketamine treatment while patients have a more positive processing bias may prolong ketamine antidepressant effects and help to reduce rates of relapse.

## 5. Conclusion

In conclusion, the findings from this exploratory study suggest that a single ketamine treatment may modulate negative affective bias. These findings add to our limited knowledge of cognitive mechanisms underlying these rapid antidepressant effects. Future larger trials are required to confirm this result.

## Figures and Tables

**Table 1:** Participant demographic and clinical information.

Variable	Control *N* = 23 M (SD)	Midazolam *N* = 11 M (SD)	Ketamine *N* = 10 M (SD)	*F*/*χ*^2^	*p*
Gender (male : female)	18 : 5	9 : 2	8 : 2	.06	.97
Age	48.4 (13.7)	49.1 (14.1)	44.7 (10.3)	.04	.70
Years of education	18.0 (4.0)	17.2 (5.1)	15.6 (3.4)	1.11	.34
Duration of current episode (months)	—	60.4 (61.9)	40.2 (16.0)		
Antidepressants (yes : no)	—	11 : 0	8 : 2		
DASS-21 Depression					
Pre	5.1 (4.1)	34.4 (6.0)^a^	36.6 (6.7)		
Post	—	30.2 (6.4)^a^	25.0 (8.9)		
DASS-21 Anxiety					
Pre	3.57 (4.6)	7.4 (10.1)^a^	9.4 (11.0)		
Post	—	6.2 (8.1)^a^	4.0 (5.8)		
DASS-21 Stress					
Pre	7.3 (5.2)	18.0 (9.4)^a^	17.6 (8.9)		
Post	—	18.2 (9.1)^a^	12.6 (8.4)		
DASS-21 Total					
Pre	16.0 (12.0)	59.8 (18.5)^a^	65.6 (22.3)		
Post	—	54.6 (14.6)^a^	41.20 (11.9)		
MADRS					
Pre	—	31.8 (5.3)	29.6 (4.1)		

Abbreviations: DASS-21: Depression, Anxiety, Stress Scale; MADRS: Montgomery Asberg Depression Rating Scale. ^a^*N* = 10.

**Table 2:** Neurocognitive measures and outcomes.

Neurocognitive measures		Control *N* = 23	Midazolam *N* = 11	Ketamine *N* = 10	Time⁣^∗^	Group⁣^∗^	Time × group^∗^
		M (SD)	M (SD)	M (SD)	*p*	*p*	*p*
SST %	Pre	9.1 (8.8)	36.8 (19.4)	50.1 (31.8)	.17	.93	**.03**
	Post	-	41.8 (20.9)	30.1 (26.9)			
EST NEB (ms)	Pre	40.4 (105.2)	-51.3 (202.8)	41.9 (131.9)	.46	.20	.45
	Post	—	39.7 (174.6)	41.3 (100.5)			
EST PEB Response time (ms)	Pre	9.1 (152.5)	-43.0 (165.8)	-0.69 (78.0)	.74	.22	.97
	Post	—	-33.6 (52.6)	10.6 (77.5)			
AGNG Neg (ms)	Pre	524.6 (54.7)	542.9 (65.4)	500.4 (79.9)	.99	.28	.62
	Post	—	536.1 (63.4)	507.7 (105.3)			
AGNG Pos (ms)	Pre	514.2 (56.6)	534.5 (56.6)	483.1 (67.5)	.23	.07	.96
	Post	—	545.6 (58.8)	495.0 (76.7)			
Ruff 2 & 7 total speed T-score	Pre	50.3 (8.8)^b^	44.00 (5.1)^a^	49.6 (4.9)^c^	**.01**	.08	.28
	Post	—	47.8 (6.4)^a^	51.2 (5.5)^c^			
COWAT total correct	Pre	41.83 (11.1)	43.5 (9.6)^a^	35.5 (6.6)	**.04**	**.02**	.25
	Post	—	48.3 (11.5)^a^	36.9 (9.0)			

Abbreviations: EST NEB: Emotional Stroop Task Negative Expressions Bias; EST PEB: Emotional Stroop Task Positive Expressions Bias; COWAT: Controlled Word Association Test; AGNG Neg: Affective Go/No Go Task Negative Shift; AGNG Pos: Affective Go/No Go Positive Shift; Ruff 2 & 7: Ruff 2 & 7 Selective Attention Test; SST: Scrambled Sentence Task; ^a^*N* = 10; ^b^*N* = 19, ^c^*N* = 9; ⁣^∗^comparison between ketamine and midazolam groups.

## Data Availability

The data used to support the findings of this study are available from the corresponding author upon request.
